# Quorum Sensing and Bacterial Social Interactions in Biofilms

**DOI:** 10.3390/s120302519

**Published:** 2012-02-23

**Authors:** Yung-Hua Li, Xiaolin Tian

**Affiliations:** 1 Department of Microbiology and Immunology, Dalhousie University, Halifax, NS B3H 1W2, Canada; 2 Department of Applied Oral Sciences, Dalhousie University, Halifax, NS, B3H 1W2, Canada; E-Mail: xiao-lin.tian@dal.ca

**Keywords:** quorum sensing, biofilms, bacterial social interactions, bacterial infections

## Abstract

Many bacteria are known to regulate their cooperative activities and physiological processes through a mechanism called quorum sensing (QS), in which bacterial cells communicate with each other by releasing, sensing and responding to small diffusible signal molecules. The ability of bacteria to communicate and behave as a group for social interactions like a multi-cellular organism has provided significant benefits to bacteria in host colonization, formation of biofilms, defense against competitors, and adaptation to changing environments. Importantly, many QS-controlled activities have been involved in the virulence and pathogenic potential of bacteria. Therefore, understanding the molecular details of quorum sensing mechanisms and their controlled social activities may open a new avenue for controlling bacterial infections.

## 1. Introduction

It was believed for many years that bacteria, unlike eukaryotic organisms, behaved as self-sufficient individuals and maintained a strictly unicellular life-style [1–3]. During infections, bacterial mass was considered nothing more than the sum of these individuals. Our perception of bacteria as unicellular life-style was deeply rooted in the pure culture paradigm of Robert Koch’s era, when Koch established his “golden criteria” to define a bacterial pathogen by using pure-culture approaches [3]. Indeed, Koch’s concept has led to the great success in the identification of bacterial pathogens and development of antibiotic treatments in acute bacterial infections [3,4]. However, pure-culture planktonic growth of bacteria rarely exists in natural environments. In fact, bacteria in Nature largely reside in a complex and dynamic surface-associated community called a biofilm [3,5,6]. If viewing an intact biofilm under microscope, one may immediately find that bacteria in biofilms do not randomly stick together, but rather form a well-organized community with numerous specialized configurations [5,6]. One may also find that bacterial cells in biofilms physically interact with each other and maintain ‘intimate’ relationships [5,6]. Even without physical contact, bacteria living at the same community likely secrete small extra-cellular molecules to interact with each other [7–10]. It was not until the last three decades that our view of self-sufficient unicellular lifestyle of bacteria has changed. The advances from at least two major research areas, biofilm development and bacterial quorum sensing, have led us to begin to appreciate, in much more detail for the first time, the concept that bacteria can organize into groups, form well-organized communities, and communicate for coordinated activities or social life that was once believed to be restricted to multi-cellular organisms [3,6–13].

Microbiologists have discovered an unexpectedly high degree of coordinated multi-cellular behaviors that have led to the perception of biofilms as “cities” of microorganisms [6]. Especially, many bacteria have been found to regulate diverse physiological processes and group activities through a mechanism called quorum sensing, in which bacterial cells produce, detect and respond to small diffusible signal molecule [7–14]. It has long been known that in infectious diseases the invading bacteria need to reach a critical cell density before they express virulence and overwhelm the host defense mechanisms before they initiate an infectious disease [1–3]. Since quorum-sensing mechanisms are widespread in both prokaryotic and single-celled eukaryotic organisms such as fungi [7–9,15], it is not surprising that cell-cell communication through quorum sensing has important implications in microbial infections. A growing body of excellent reviews has highlighted quorum sensing and its roles in bacterial social activities, biofilm formation and infectious diseases over the last decades [3–17]. A connection between quorum sensing and microbial biofilms has brought together investigators who have a common interest in how bacteria function as a group for social activities. The integration of scientists who are interested in bacterial social behavior into biofilm research field represents a powerful force in the development of new strategies to prevent and treat biofilm-associated infections [12,13,16] and other public health problems in food industry, agriculture and environmental protection [1–3,13].

## 2. Bacterial Social Interactions in Biofilms

Most surfaces on this planet teem with microbial biofilms that account for over 99% of microbial life [3,6,13]. Biofilms are spatially structured communities of microbes whose function depends on a complex web of symbiotic interactions [3,6]. High cell density and close proximity of diverse species of microorganisms are typical of life in natural biofilms, where organisms are involved in complex social interactions that occur both within and between species and can be either competitive or cooperative [3,17–22]. Competition for nutrients and other growth parameters is certainly an important driving force for the development of biofilm structure. Increased cell density favors chemical signals to communicate with the responding cells for social interactions in biofilms, likely adding another level of complexity to biofilms [17–20]. Furthermore, the expression of different adhesins, their cognate receptors, and exopolymeric components by individual cell types within a biofilm community can contribute to overall biofilm development [3,6,19,20]. In particular, many bacteria are capable of using a quorum sensing mechanism to regulate biofilm formation and other social activities [12–14,18]. Under such complex conditions, bacteria could benefit from division of labor, collective actions, and other forms of cooperative activities with their neighbors [17–20]. For example, dental plaque is a well-recognized biofilm community characterized by its vast biodiversity (>700 species) and high cell density (10^11^ cells/g wet wt) [5,14,17,19]. The high cell density and species diversity within dental biofilms coupled with environmental fluctuations should create an environment that is conducive to inevitable intra- and inter-species interactions [19]. Indeed, cooperative interactions among oral bacteria have been well studied, including bacterial coaggregation that facilitates coadhesion of bacterial pairs to the tooth surface, nutritional synergy and complementation to enable cell growth in saliva, and formation of food chains through metabolic cooperation between two or more species [5,17]. These cooperative interactions probably play very important roles in the development of dental biofilms [17]. In addition, competition or antagonistic interactions among different species may be equally important for the maintenance of a balance relationship between microbes in dental biofilms, and between dental biofilms and the host defense mechanisms in the oral cavity [5,17,19]. Many bacteria in dental biofilms produce peptide bacteriocins, which may play important roles in inter-species competition, biodiversity and ecological fitness of microbes [19,21–23]. Numerous studied have shown that production of bacteriocins by naturally transformable streptococci, such as *S. mutans*, *S. gordonii*, *S. sanguinis* and *S. mitis* is tightly controlled by a quorum sensing system that also regulates genetic competence and biofilm formation in these species [21–23]. Interestingly, all of these species are considered as primary colonizers in dental biofilms, although bacteriocins produced by one species kill other species [17,19]. These sophisticated interactions represent good examples of microbial social activities in natural microbial biofilms. These social activities may play important roles in balancing competition and coexistence of these organisms within a microbial community like dental biofilm, maintaining biodiversity and homeostasis of microbes in the same ecosystem [19,20,24].

## 3. Problems from Bacterial Social Activities

Since the discovery of penicillin in 1928, antibiotics have proven tremendously successful in controlling acute bacterial infections [1–4]. Microbiologists have learned to predict antibiotic effects *in vivo* by evaluating the minimal inhibitory concentration (MIC) or the minimal bactericidal concentration (MBC) *in vitro*. MIC and MBC assess the effect of antibiotics against planktonic organisms in exponential growth phase, therefore, correctly predicting antibiotic efficacy against rapidly dividing bacteria in acute infections. However, clinicians who deal with chronic biofilm-associated infections, such as medical device or implant infections, frequently fail to cure their patients by using the same treatments [1–3]. There is increasing evidence that biofilm infections often resist to the highest deliverable levels of antibiotics [3,6]. The infections may persist for months or years, resulting in long-term suffering and tissue damage [1,2]. There are many examples of biofilm infections threatening the human health, including infections of bone, airway/lung tissue, cardiac tissues, middle ear, gastrointestinal tract, eye, urogenital tract, prosthetic devices, indwelling catheters, implants and dental diseases [1–3,19]. As reported by the Center for Disease Control and Prevention (CDC), biofilms have been involved in over 65% of hospital infections [3]. Because of inherent resistance to antibiotics, biofilm infections can be life threatening to immuno-compromised patients [1–3].

Studies of microbial biofilms have led to realize that bacteria present in biofilms have characteristics different from those of the free-living counterparts, including a significantly increased resistance to antibiotics and the host immune response [1,2,6]. Living in biofilms allows bacteria to have several advantages to interact with each other and function as a group for coordinated activities. Bacteria with altered physiological activities (biofilm phenotypes) are known to result largely from bacterial social activities controlled by quorum sensing or cell-cell interaction via direct contact in biofilms [12–15,17–20]. More importantly, these changed phenotypes are usually associated with the virulence and pathogenicity of bacteria [24,25]. In modern clinical microbiology, the establishment of bacterial biofilms has been considered an important pathogenic trait in many chronic infections [1–3,19,24,25]. For example, medical device- or implant-associated biofilm infections are clinically characterized by chronic and persistent processes [1–3]. These infections are resistant to the highest deliverable levels of antibiotics, often resulting in significant tissue damage and long-term suffering of patients [1–4]. The armament of therapeutic agents available to treat these infections today takes little account into the unique biology of bacterial social interactions in biofilms. This becomes a problem, because biofilms resulting in persistent infections cannot be resolved with standard antibiotic treatments. Since we have not considered the problem of bacterial group behaviors until recently, effective therapeutic strategies to prevent or treat biofilm infections are currently not available. Therefore, understanding bacterial social behaviors and their molecular mechanisms in the development of biofilms will greatly facilitate the development of novel strategies in the prevention and treatment of biofilm infections.

## 4. Quorum Sensing as a Central Mechanism to Regulate Bacterial Social Activities

Bacteria in a community may convey their presence to one another by producing, detecting, and responding to small diffusible signal molecules called autoinducers [7–11]. This process of intercellular communication, called quorum sensing, was first described in the marine bioluminescent bacterium *Vibrio fischeri* [7–9,11,26]. *V. fischeri* lives in symbiotic associations with a number of marine animal hosts. In these partnerships, the host uses light produced by *V. fischeri* for specific purposes such as attracting prey, avoiding predators, or finding a mate [7–9,11,26]. In exchange for the light it provides, *V. fischeri* obtains a nutrient-rich environment where it resides. A luciferase enzyme complex is found to be responsible for light production in *V. fischeri*. Bioluminescence occurs only when *V. fischeri* is at high cell density, which is controlled by quorum sensing [26]. Specifically, the production and accumulation of, and the response to, a minimum threshold concentration of an autoinducer regulate density-dependent light production in *V. fischeri*, and enables *V. fischeri* to emit bioluminescence light [7–9,26]. Remarkably, such a quorum sensing-mediated social activity for light emission by marine bacteria has been found at a global scale [27]. Over the centuries, mariners have reported witnessing mystery nocturnal displays, where the surface of the sea produces an intensive, uniform and sustained glow, called “milky sea”, which extends horizontally over a hundred kilometers of sea surface. By using a satellite sensor system, Miller and colleagues detected such massive bioluminescence emission of a “milky sea” in the northwestern Indian Ocean [27]. The “milky sea” is an excellent manifestation of quorum sensing-mediated bioluminescence bloom produced by massive numbers of a marine bacterium, *V. harveyi*, living in association with microalga colonies on the surface of the sea [7,26,27]. Recent studies have well documented that such a global scale of bacterial social activities for bioluminescence glowing is tightly regulated by multiple quorum sensing pathways that form a complex regulatory network [7,9,26,27].

It is now known that many bacteria regulate their social activities and physiological processes through a quorum sensing mechanism, including symbiosis, formation of spore or fruiting bodies, bacteriocin production, genetic competence, programmed cell death, virulence and biofilm formation (Table 1). The processes controlled by quorum sensing are diverse and reflect the specific needs of particular communities. In many bacteria, quorum sensing represents a central mechanism to regulate social activities, allowing bacteria to reap benefits that would be unattainable to them as individual cells [7–9,26]. Increasing evidence shows that quorum sensing-mediated social activities favor microbial interactions and are believed as major mechanisms to regulate population-level virulence of bacteria [12–14,24,28–32]. These studies have produced important insights into the social biology of microbes in biofilms and in bacterial infections.

## 5. Common Themes in Bacterial Quorum Sensing

Quorum sensing relies upon the interaction of a small diffusible signal molecule with a sensor or transcriptional activator to initiate gene expression for coordinated activities [7–11,26]. Quorum sensing systems in bacteria have been generally divided into at least three classes: (1) LuxI/LuxR–type quorum sensing in Gram-negative bacteria, which use acyl-homoserine lactones (AHL) as signal molecules; (2) oligopeptide-two-component-type quorum sensing in Gram-positive bacteria, which use small peptides as signal molecules; and (3) *luxS*-encoded autoinducer 2 (AI-2) quorum sensing in both Gram-negative and Gram-positive bacteria. Each type of signal molecule is detected and responded by a precise sensing apparatus and regulatory network [7–11,26].

**Figure 1.**
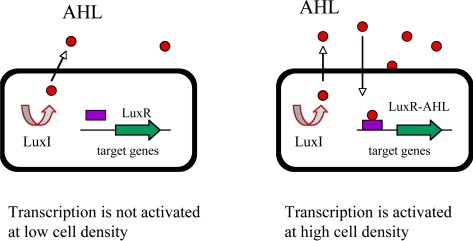
The LuxI/LuxR–type quorum sensing in Gram-negative bacteria. The LuxI-like protein is an autoinducer synthase that catalyzes the formation of a specific acyl-homoserine lactone (AHL). The AHL freely diffuses through the cell membrane at high cell density. The LuxR is a transcriptional regulator that binds to the diffusing AHL and in turn activates the transcription of its target genes.

In Gram-negative bacteria, signal molecules are acyl-homoserine lactones (AHL) whose synthesis is dependent on a LuxI-like protein [28–30]. AHLs freely diffuse across the cell membrane and increase in concentration in proportion to cell density. A cognate LuxR-like protein is responsible for recognition of the AHL and when bound to the AHL, LuxR-like protein binds to specific promoter DNA elements and activates transcription of target genes (Figure 1). The biochemical mechanism of action of the LuxI/LuxR pairs is conserved. The LuxI-like enzymes produce a specific AHL by coupling the acyl-side chain of a specific acyl-acyl carrier protein (acyl-ACP) from the fatty acid biosynthetic machinery to the homocysteine moiety of *S*-adenosylmethionine (SAM). This intermediate lactonizes to form acyl-HSL, releasing methylthioadenosine [28,29]. There are hundreds of Gram-negative bacteria identified to use LuxI/LuxR-type quorum sensing to control a wide range of cellular processes. Each species produces a unique AHL or a unique combination of AHL and, as a result, only the members of the same species recognize and respond to its own signal molecule [7,8,25,28]. Many other examples of Gram-negative circuits exist that utilize a LuxI/LuxR-type quorum sensing mechanism onto which additional regulatory factors have been layered [7–9,25–30].

**Figure 2.**
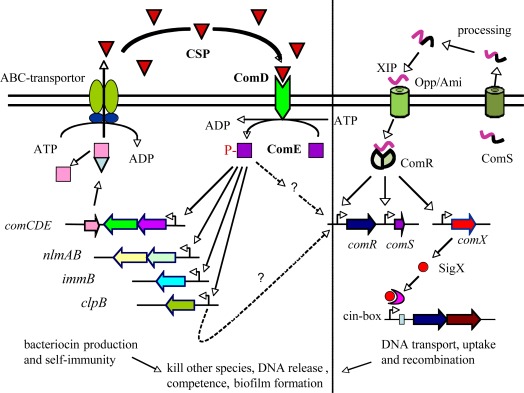
A schematic diagram indicating two types of signaling peptide-mediated quorum-sensing systems in Gram-positive bacterium, *S. mutans*. The ComCDE quorum-sensing system primarily regulates production of bacteriocins and bacteriocin self-immunity proteins, while the newly identified ComRS quorum-sensing system proximally controls competence development via the control of *sigX* that encodes an alternative sigma factor, SigX (ComX). CSP is ComC signal peptide; XIP is mature *sigX*-induced peptide. Opp/Aml is an ABC transporter (peptide importer).

In contrast to those in Gram-negative bacteria, there are two types of quorum-sensing systems identified in Gram-positive bacteria [10,31–37]. In the first type, quorum-sensing systems generally consist of three components (Figure 2), a signaling peptide known as autoinducing peptide (AIP) and a two-component signal transduction system (TCSTS) that specifically detects and responds to an AIP [7,10,26,31,32]. In further contrast to AHL signals, cell membrane is not permeable to AIP but rather a dedicated oligopeptide transporter, largely an ABC transporter, is required to secrete AIP into the extracellular environment [10,31,32]. Gram-positive bacteria normally produce a signal peptide precursor, which is cleaved from the double-glycine consensus sequence and the active AIP is then exported through a peptide-specific ABC transporter into their environments. Most of signaling peptides in Gram-positive bacteria typically consist of 5–25 amino acids and some contain unusual side chains [31,32]. Detection of signaling peptides in Gram-positive bacteria is mediated by a two-component signal transduction system, which consists of a membrane-associated, histidine kinase protein sensing the AIP, and a cytoplasmic response regulator protein enabling the cell to respond to the peptide via regulation of gene expression [10,26,31,32].

In recent years, the second type of quorum sensing system has been identified in several groups of Gram-positive streptococci, including those in the salivarius, pyogenic, mutans and bovis groups [33–35]. This new system is called ComRS, which involves sensing a small double-tryptophan signal peptide pheromone, XIP, inside the cells after its internalization by an oligopeptide ABC transport system Opp/Ami [33]. Once internalized, the XIP pheromone interacts with a transcriptional regulator, ComR, proximal regulator of *sigX* that encodes a master regulator or alternative sigma factor SigX (ComX), in turn activating later competence genes for genetic transformation [33–35]. Interestingly, *S. mutans* has been found to possess both ComCDE and ComRS quorum sensing systems that regulate bacteriocin production and genetic competence, respectively (Figure 2; [33]). Studies of subpopulation-specific transcriptome analyses in *S. mutans* suggest that a high level of ComE may induce a positive feedback loop for ComED and further activate ComR and SigX through an unknown mechanism either directly or indirectly, therefore, activating genetic competence and programmed cell lysis [36,37].

In addition to the above quorum-sensing mechanisms, another type of quorum sensing, called autoinducer 2 (AI-2), has been described in both Gram-negative and Gram-positive organisms [7,8,11]. Different from the above quorum-sensing systems that are specifically for intra-species signaling, AI-2 allows for inter-species communication, so it is called a “universal language” used for cross-species communication [11,26]. AI-2, which was first characterized in a marine bacterium *V. harveyi*, is a furanosyl borate that regulates cell density-dependent bioluminescence [38,39]. The synthesis of AI-2 depends on a *luxS* encoded synthase, which is a metabolic enzyme involved primarily in the conversion of ribosyl-homocysteine into homocysteine and 4,5-dihydroxy-2,3-pentanedione (DPD), the precursor of AI-2 [38]. The LuxR protein is a cytoplasmic receptor and also functions as a transcriptional activator [11,26,39]. A *luxS* mutation interrupting this metabolic pathway changes the whole metabolism of the bacteria. The homologues of LuxS have been found in many species of bacteria, suggesting that AI-2 quorum sensing is very prevalent among prokaryotes [11,39]. With such a wide distribution, it is not surprising that the *luxS*-encoded quorum sensing mechanism has important roles in microbial ecology. The discovery of AI-2 that is produced and detected by a large number of diverse bacteria implies that bacteria have a means to assess the cell density of other species in a microbial community, facilitating interspecies communication and social interactions among species in the community [7,8,11,39].

## 6. Quorum Sensing in the Regulation of Biofilm Formation and Virulence

In 1998, Greenberg and his colleagues first described the role of the *las* quorum sensing in biofilm formation of *Pseudomonas aeruginosa* [40], a Gram-negative bacterium that is considered as one of the most common opportunistic pathogens in human infections causing fatal systemic disease under certain conditions [41–43]. Lung infections with biofilms of this pathogen are particularly common in patients with cystic fibrosis [41,42]. In this organism, quorum sensing is highly complex and consists of two interlinked *N-*acyl homoserine lactone-dependent regulatory circuits, which are modulated by many regulators acting both at transcriptional and post-transcriptional levels [40,44]. These researchers found that the *lasI* mutant defective in the production of the autoinducer 3-oxo-C12-HSL formed biofilm cell clusters that were 20% of the wild-type biofilm in thickness and were sensitive to detergent removal. When the 3-oxo-C12-HSL was added to the system, the *lasI* mutant was once again able to form structured biofilms [40]. This finding suggests that quorum sensing plays an important role in the development of biofilms, and more importantly, it makes an inextricable connection between quorum sensing and biofilm formation. Subsequent studies further show that the quorum-sensing circuits in *P. aeruginosa* orchestrate a symphony of several virulence factors, such as exoproteases, siderophores, exotoxins and rhamnolipids [41–43], In particular, the QS-controlled virulence expression in *P. aeruginosa* has been demonstrated both *in vitro* and *in vivo* model systems [40–42].

Gram-positive bacteria, such as *Staphylococcus aureus* and various streptococci, use signal peptide-mediated systems for quorum sensing [14,31]. For example, *S. aureus* is a leading cause of nosocomial infections worldwide and causes diseases from mild skin infections to potentially fatal systemic disorders [31,45,46]. Many infections caused by *S. aureus*, such as endocarditis, osteomyelitis and foreign-body related infections are not caused by free-living cells but rather by biofilms [45]. Many virulence factors involved in staphylococcal infections, including surface-associated adhesins, hemolysin, toxins and autolysins, are regulated by quorum sensing via the accessory gene regulator (*agr*) system [31,45–47]. The *agr* locus in *S. aureus* consists of two divergent transcription units that are transcripted under control of the promoters P2 and P3, respectively. The P2 operon consisting of *agrBDCA* encodes four proteins that constitute the Agr-sensing mechanism. The autoinducer (AIP) molecule from *S. aureus* is an octapeptide with a unique thioester ring structure, which is generated from its precursor, AgrD, and secreted out of the cell through the action of the AgrB membrane protein. As its concentration increases in extracellular environment, AIP binds to the histidine kinase receptor, AgrC, resulting in its autophosphorylation. Phosphorylated AgrC in turn activates the response regulator AgrA, which functions cooperatively with another global regulator, SarA, to drive the transcription at the P2 and P3 promoters, resulting in elevated intracellular levels of RNAII (QS amplification) and RNAIII (exoproteins) [7–9,31]. Interestingly, AIP from one *S. aureus* strain is not only capable of activating the *agr* regulon in itself, but also inhibits the *agr* activation of other strains (46). Sequence variation analysis of *agrB*, *agrD* and *agrC* has led to identification of at least four *S. aureus agr* specificity groups, in which AIP produced by one group inhibits *agr* expression in other groups [31,46]. Such cross-strain inhibition of the *agr* response has been exploited for the treatment of staphylococcal skin abscess in animals [47]. There is mounting evidence that the *agr* phenotype and expression patterns may influence several aspects of biofilm phenotypes, including attachment of cells to surfaces, biofilm dispersal, and even the chronic nature of biofilm-associated infections [45–47]. The *agr* quorum-sensing system increases the expression of many secreted virulence factors in the transition from late-exponential growth to stationary phase [31]. The importance of the *agr* system for the development of invasive infections and disease progress has been demonstrated in several infection models, such as subcutaneous abscesses, murine arthritis or pneumonia, rabbit osteomyelitits or endocarditis [31,45–47].

*Streptococcus mutans* is another good example of bacteria that use quorum sensing to regulate social activity, biofilm formation and virulence [14,48–51]. *S. mutans* is a bacterium that has adapted a biofilm life-style for survival and persistence in its natural ecosystem, dental plaque [19,48]. Under appropriate conditions, however, *S. mutans* can rapidly produce acids from dietary fermentable carbohydrates and initiate demineralization of the tooth surface or dental caries [50,51]. *S. mutans* is therefore considered an important etiological agent of dental caries. *S. mutans* has a well-conserved quorum-sensing system that consists of at least three gene products encoded respectively by *comCDE* (Figure 2). The *comC* encodes a signal peptide precursor, which is cleaved and exported to release a 21-amino acid signal peptide (ComC signal peptide or CSP) through a peptide-specific ABC transporter encoded by *cslAB*. The *comDE* genes encode a two-component transduction system (TCSTS) that specifically senses and responds to CSP [36,48–52]. When it reaches a critical concentration, the CSP interacts with ComD histidine kinase receptor of neighboring cells and results in autophosphorylation of the ComD in expense of ATP. The ComD activates its cognate response regulator, ComE, through phosphorylation and in turn activates its target genes, presumably *comDE* and genes encoding numerous QS-dependent bacteriocins and bacteriocin self-immunity proteins [22,23,36,37]. This signaling cascade also triggers DNA release and genetic competence [53–55]. Recently, a new quorum sensing system with a double-tryptophan peptide pheromone as signal molecule has been identified in *S. mutans* [33]. Interestingly, this new system appears to intersect with the ComCDE signal transduction pathway and directly controls ComRS and an alternative sigma factor, SigX (ComX), to activate competence development of a subpopulation for genetic transformation [36].

Perhaps, the most fascinating finding in *S. mutans* is that ComCDE quorum-sensing system appears to connect to bacteriocin production, stress response, genetic competence and biofilm formation, the key virulence factors in the pathogenesis of this organism [23,48–51]. Studies showed that the biofilms formed by the ComC mutant that did not produce CSP had a changed biofilm phenotype with reduced biomass; conversely, adding synthetic CSP into the culture restored the wild-type biofilm [49]. Interestingly, the increased biomass could be abolished in the presence of DNase I, an endonuclease that cleaves double-stranded DNA, suggesting a role of the quorum sensing system in regulating DNA release and biofilm formation [22,54]. In fact, studies of *S. pneumoniae* have shown that CSP-mediated competence induces a programmed cell lysis of a subpopulation of *S. pneumonia* along with the release of DNA from lysed cells [32,55,56]. This phenotype, called fratricide, is suggested to be an important mechanism to ensure that competence cells obtain available DNA during genetic transformation [32,55]. Recent studies show that the *S. mutans* CSP also triggers programmed cell lysis and DNA release from a small subpopulation and a CSP-induced bacteriocin (CipB) appears to be responsible for such cell lysis [37,53]. *S. mutans* has long been known to produce an array of bacteriocins and bacteriocin-immunity proteins, including *nlmAB* encoding mutacin IV, *smB* encoding a class I bacteriocin and *bip* encoding a bacteriocin immunity protein [21–23]. In addition, deletion of *comCDE* genes resulted in attenuation of virulence and cariogenic potential of *S. mutans* in a rat caries model, suggesting that this quorum sensing system plays a role in the pathogenesis of *S. mutans* in dental caries [49,51]. By using different *in vivo* model systems, Oggioni *et al.* also demonstrated that the QS-controlled competence regulon in S*. pneumoniae* played important roles in the pneumococcal pathogenesis, involving two types of QS-controlled gene expression profiles that were corresponding to two types of pneumococcal infections from bacteraemic sepsis (planktonic-like state) to tissue infections, pneumonia or meningitis (biofilm-like state) [57]. These studies may provide an ample explanation for the connection between quorum sensing, biofilm formation and their pathogenic potential.

Autoinducer 2 (AI-2) mediated-quorum sensing mechanism is widely distributed in bacterial species [7–9,11]. AI-2 has also proved important in the development of structured biofilms, especially multi-species biofilms in natural ecosystems [13,17,58,59]. For example, AI-2 levels in a mixed culture of *Actinomyces naeslundii* T14V and *S. oralis* 34 are critical to the duel-species phenotypes of mutualistic interdigitated biofilm growth of these two organisms when saliva is used as the sole nutrient source [58]. A duel-species biofilm containing an *S. oralis* 34 *luxS* mutant and *A. naeslundii* T14V does not show mutualistic interdigitated growth, but this defect can be restored by adding synthetic DPD, a product of the LuxS enzyme, into the culture and such a complementation is concentration-dependent. However, the mechanism behind this connection between AI-2 activity and biofilm formation is not well understood. Trappetti and colleagues have recently demonstrated that *luxS* appears to be a central regulator to mediate iron-dependent biofilm formation, competence and fratricide in *S. pneumoniae* [60]. The LuxS-dependent biofilm formation and its molecular mechanism have been further demonstrated in clinical isolate of *S. pneumoniae* D39 [61]. Many species of bacteria in natural biofilms like dental plaque have been found to have a *luxS* homolog in their genomes [58,59,62]. They may play important roles in inter-species communication and biofilm formation, although the mechanisms how these systems mechanically work in microbial communities remains to be studied.

## 7. Quorum Sensing and Social Activities on Bacterial Ecology and Evolution

Most microbes live in populations and rely on population-level traits for their survival and physiological activities [12,13]. Also, bacteria achieve strength in numbers by collectively secreting virulence factors required for pathogenesis [63]. An increasing body of evidence suggests that quorum sensing-mediated social activities among a clonal population are common in bacteria [64]. During quorum sensing, bacterial cells cooperate to obtain group-specific benefits [64–67]. One of the best-described examples is that *Mycococcus xanthus* requires cell-cell signaling and social cooperation to form a fruiting body containing hardy spores in response to starvation [68]. Through social cooperation, a portion of the population survives to starvation by forming the fruiting bodies, but most cells in the population, which provide the cooperation, are sacrificed. This phenomenon is called altruism, in which cooperation benefits the group but cost for the cooperating individual [66,68]. It has been found that such quorum sensing-controlled cooperation is widespread in many bacteria (Table 1), providing a bacterial population with group-derived benefits or altruism. The idea of quorum sensing and its controlled social activities that provide altruism has gained wide acceptance in recent years.

**Figure 3.**
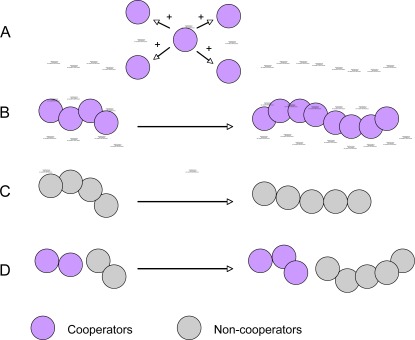
A schematic diagram describing quorum sensing-mediated social cooperation and conflict. Social cooperation provides benefits to the population but has a cost for the cooperative cells. Cooperative cells provide fitness benefits to the entire population (**A**) and have a higher productivity or yield in an exoproduct (**B**). However, non-cooperative cells (cheaters) may have the lower productivity (**C**), but can exploit the benefits from the cooperative cells without contribution in mixed populations (**D**).

From an evolutionary point of view, however, bacterial social behavior may create conflict of interest and even potential risk to the population, because evolutionary theory predicts that individuals that cooperate can be exploited by selfish individuals or “cheaters” that do not cooperate but obtain the benefit from cooperators [65–67,69]. The advantage of cooperation is easy to understand if populations are monoclonal and the fitness cost to individual cells is outweighed by the benefit to the population. However, in many realistic situations microbial populations are not monoclonal but rather heterogeneous populations where cooperators and non-cooperators interact. Cooperation provides fitness benefits or “public goods” to the population, but the population benefits often come at a cost to individuals [65–70]. The question then arises as to why individuals produce costly public goods if these increase the fitness of other individual at their own cost. This is a conflict of interest between the fitness of individuals and the fitness of the group. The basis for explanation of such cooperation is provided by Hamilton’s inclusive fitness or Kin selection theory, which states that cooperation evolves between genetically related individuals or relatives [66]. A good illustration of this conflict is the trade-off between slow growth rates with a high yield versus fast but wasteful growth. Higher yields make a more economic use of limited resources and, therefore, can be beneficial to the entire population (Figure 3). The population benefit comes at the expense of individual-level restraint, as cells could grow faster with lower yields [67]. Another example is the persister phenotype, which has a role in bacterial antibiotic resistance. Persisters are cells in a dormant state that typically compose a small fraction of all cells in a population. As many antibiotics act on growing cells, dormant cell can resist short treatments and afterwards revert back to active growth to restore the population. The persister phenotype is therefore a bet-hedging strategy that confers antibiotic resistance, but does so at the cost of the growth by entering the dormant state [67]. The emergence of non-cooperators through mutation is a major challenge to cooperative phenotypes. Diggle *et al.* observed this effect in quorum-sensing populations of the opportunistic pathogen *P. aeruginosa* [65]. They found that quorum sensing provided a benefit at the group level, but exploitative individuals could avoid the cost of producing the QS signal (signal negative mutant) or of performing the cooperative behavior that was coordinated by QS (signal-blind mutant). These non-cooperators can therefore spread in the population. These researchers also showed a solution to this problem of exploitation by kin selection, which might be highly important in microbial social behaviors because of their clonal reproduction and relatively local interactions [65]. Natural biofilms in many environments are often characterized by high cell density and high diversity of microbial species. The biofim community allows close cell-cell interactions within or between species, resulting in inevitable intra- and inter-species interactions, including both cooperation and competitions [17–21]. These interactions may play very important roles in maintaining homeostasis of microbes in a biofilm community [17,19]. The diversity and interactions that can arise in biofilms represent unique opportunity for testing ecological and evolutionary theories.

## 8. How Might Quorum-Sensing Signal Molecules Function in Biofilms?

To date, almost all quorum-sensing mechanisms described have been studied in the context of planktonic cultures. This is understandable because it simplifies the signaling process. In liquid cultures, all bacteria are presumed to be physiologically similar and are producing signal molecules at the same rate. However, quorum sensing and signal transduction in biofilms might be much more complicated because of a range of physical, chemical and nutritional factors that may influence signal production, stability, distribution and efficiency to interact with their cognate receptors in a biofilm. How quorum sensing signal molecules function in a biofilm and how frequently quorum sensing is activated in a biofilm are largely unknown. Bacterial biofilms normally consist of bacterial cells and an extracellular matrix, including a mixture of secreted proteins, polysaccharides, nucleic acids and dead cells [1–3]. AHL molecules are known to diffuse freely across the cell membrane, so that they are assumed to have little problem to reach their target receptors via free diffusion in the biofilm matrix [12,13,28]. However, signaling peptides produced from Gram-positive bacteria are likely influenced by physical, chemical and biological factors within a biofilm because of the feature that small peptides likely interact with charged molecules [10,45]. Currently, little is known about whether signal peptides can be affected by diffusion limitation or by non-specific binding to polysaccharides, proteins, DNA and even cell wall components within the biofilm. In addition, the cost for a Gram-positive bacterium to produce an active signal peptide is very expensive process. Keller and Surette have estimated that the production of a signal peptide in *S. aureus* costs 184 ATP but only 8 ATP for an AHL in *P. aeruginosa* [70]. Clearly, the cost for production of a signal peptide is much more expensive in Gram-positive bacteria. It is therefore reasonable to assume that nutrient or energy source will be significant factors to influence signal peptide-mediated quorum sensing and activities in Gram-positive biofilms.

Theoretically, signal molecules that function to estimate population density could be affected by the concentration of a signal molecule, diffusion limitation, accessibility to the receptor, degradation and production of the same autoinducer such as AI-2 by third parties, whether intentionally or by chance. Some workers have used mathematic models to estimate the potential influence and possible mechanisms behind [71,72]. Quorum sensing could be considered as diffusion sensing (DS), since QS activation depends on the diffusion of a signal molecule to and interact with the cognate receptor [70]. This implies that QS is an autonomous activity of single cells to detect mass-transfer limitation. However, the QS and DS concepts may encompass an evolutionary conflict. Quorum sensing postulates that bacteria sense their density to allow them to engage in social behavior; accordingly, quorum sensing assumes that sensing evolved because of the group benefits [64–69]. In contrast, DS assumes that sensing evolved because of a direct fitness benefit for the individual cells, so it does not invoke group benefits for the evolution of autoinducer sensing. By unifying these conflicting concepts, Hense *et al.* [71] proposed a new concept of efficiency sensing (ES), in which some of the problems associated with signaling in complex environments as well as the problem of maintaining honesty in signaling, could be avoided when the signaling cells grow in microcolonies or in biofilms [71,72]. Using a mathematical model, these authors suggest that the spatial distribution of cells may be more important than their density, and that spatial distribution and density should be independently measured. As a consequence, efficiency sensing is a functional hypothesis that acknowledges the fact that autoinducers can measure a combination of cell density, diffusion limitation and spatial distribution of autoinducer. ES is also a unifying evolutionary hypothesis as it argues that quorum sensing have been favored by both individuals and group benefits. This new theory has described a typical mode of biofilm growth and formation of clonal clusters, but avoids the problems of complexity and cheating that autoinducer-sensing bacteria encounter *in situ*, although this model remains to be experimentally tested.

## 9. Quorum Sensing as a Novel target for Anti-Virulence Therapies

Quorum-sensing systems of bacteria rely on signal molecules and their cognate receptor proteins for gene regulation and coordinated activities [7–12]. Any compound that prevents production of signal molecules or interactions between signal molecules and cognate receptor proteins might block bacterial quorum sensing and its gene expression. The discovery of bacterial quorum sensing mechanisms has led to identification of some compounds or enzymes that quench quorum sensing, called QS interference [43,73,74]. Evidence has accumulated that such QS interference can be developed as promising approaches to control biofilm formation and microbial infections. Interestingly, anti-quorum sensing compounds exist in nature. Both plants and algae produce compounds that mimic quorum-sensing signals of many bacteria, so that they interfere with bacterial quorum sensing and its controlled activities. For example, the red seaweed called *Delisea pulchra* (Greville) that grows under the sea around Australia, produces a range of biologically active furanones [2,75]. These natural compounds are found to be powerful signal antagonists for prevention against bacterial colonization by interfering with acyl-HSL signaling systems [2,75]. This biological understanding has led to the application of furanones as inhibitors of bacterial fouling. Furanones inhibit bacterial colonization and biofilm formation through interference with acyl-HSL quorum-sensing pathway in Gram-negative bacteria [75]. They also interfere with AI-2 signaling systems in both Gram-negative and -positive bacteria. Additionally, furanones inhibit the expression of bacterial exo-enzymes that actively degrade components of the immune system, thereby, enhancing the immune response [73,75]. There is growing evidence that bacterial quorum sensing systems are involved in cross-kingdom signaling with eukaryotic organisms [8,15]. Likewise, eukaryotes are capable of actively responding to the presence of these signal molecules and produce compounds interfering with bacterial quorum sensing by acting as agonists or antagonists.

Mankind fights bacterial infections by using antibiotics or antimicrobial agents. The success of this treatment is largely based on antibiotics or antimicrobial agents that aim to inhibit bacterial growth. The major concern of this approach is the frequent development of antibiotic resistance [4]. Furthermore, a big obstacle in fighting bacterial infections is that bacteria in nature and in the human body are predominantly associated with surfaces and form biofilms, which enables bacteria to resist inhibition or removal by the highest deliverable levels of antibiotic agents [1–3]. As we began to gain control over epidemic infectious diseases, biofilm infections came to the fore. A global concern has emerged that we are entering a post-antibiotic era with a reduced capability to combat persistent biofilm infections. Because of refractory to antibiotics, biofilm infections can be life threatening to immuno-compromised patients [1–4]. Given many bacteria that employ quorum-sensing mechanisms in controlling virulence, pathogenicity and biofilm formation, quorum sensing constitutes a new target for the development of antibacterial agents with potential application in many fields. Currently, at least four strategies aiming at interference with quorum sensing have been proposed, including (1) inhibition of signal generation; (2) interference with signal dissemination; (3) blocking signal receptors; and (4) inhibition of signaling response system [43,73–75]. The key of these strategies is to interfere with bacterial quorum sensing and its controlled pathogenic activities. Knowing the molecular details of communication systems and their control of virulence and pathogenicity opens a new avenue for controlling microbial infections. The development of signal analogs that specifically block or override the bacterial command line will enable us to control the unwanted activities without affecting bacterial growth. A major difference of these compounds from antibiotics is that they do not directly inhibit bacterial growth or kill bacteria so that there is no strong selection pressure to create resistant microbes. Compounds that can inhibit signals of quorum sensing systems can be developed into potent antagonists against infectious bacteria, although there may be a risk for inactivation of antagonists. Such novel drugs that specifically target quorum sensing systems are capable of attenuating bacterial infections in a manner that is less likely to result in the development of resistant mutants [75,76]. Several studies have recently described the application of AHL analogs or signal peptide analogs to achieve inhibition of quorum-sensing circuits in some bacteria [74–76]. Zhu and Lau have recently reported a competence-stimulating peptide analog, CSP1-E1A, which inhibits competence development and reduces expression of pneumococcal virulence factors, such as choline binding protein D and autolysin A *in vitro* [77]. This peptide analog also reduces mouse mortality after lung infection and attenuates the acquisition of an antibiotic resistance gene and a capsule gene *in vivo* [77]. In addition, quorum sensing inhibitors (QSI) have been found to increase the susceptibility of bacterial biofilms to existing antibiotics both *in vitro* and *in vivo*, thereby, increasing the success of antibiotic treatment of biofilm infections [78]. For instance, a QSI that target the acyl homoserine lactone-based QS systems can increase the efficacy of conventional antibiotics, such as tobramycin, against biofilms consisting of *P. aeruginosa* and *Burkholderia cepacia* both *in vitro* and *in vivo* [78]. These studies have generated substantial knowledge about quorum sensing interference as a new antimicrobial strategy.

## 10. Concluding Remarks

In the past decade, significant advance has been made regarding bacterial quorum sensing and group behaviors. Quorum sensing is emerging as an integral component of bacterial global gene regulatory networks responsible for bacterial adaptation in biofilms. The discovery of the widespread use of quorum sensing systems in bacteria is pivotal in guiding researchers to study bacterial multicellular behaviors rather than the previous emphasis on individual cell biological processes. However, research on how bacterial quorum sensing works mechanistically in biofilms remains in their infancy. A clear challenge facing the field is to determine what factors of a biofilm influence the onset of quorum sensing and subsequent gene expression. Another key challenge is to determine functional consequences of quorum sensing in multi-species biofilms. Future research will clearly address these questions in the emerging field of bacterial social behaviors. The answer to these questions will undoubtedly provide new insights and surprises.

## Figures and Tables

**Table 1.** Examples of Bacterial Quorum Sensing Systems and their Controlled Social Traits

**Microrganism**	**Major Signal Molecules**	**Regulatory System**	**Group-Derived Benefits**	**References**
*Bacillus subtlis*	ComX CSF (PhrC) PhrA,-E, -F, -K, -H	ComP/ComA Rap proteins	Competence, sporulation, biofilm formation, antibiotic production,	[7–10,32]
*Myxococcus xanthus*	A-signal C-signal	SasSRN	Fruiting body formation or sporulation	[7–10]
*Pseudomonas aeruginosa*	3O-C12-HSL C4-HSL	LasI/LasR RhlI/RhlR OscR (orphan)	Structured biofilm formation, virulence factors	[7–10, 28–30]
*Staphylococcus aureus*	AIP-I, AIP-II, AIP-II, AIP-IV	AgrC/AgrA	Biofilm formation, virulence factors	[7–9,31]
*Streptococcus mutans*	CSP (ComC) XIP (ComS)	ComD/ComE ComR	Bacteriocins, biofilm formation, competence	[33–36]
*Streptococcus pneumoniae*	CSPs	ComD/ComE	Competence, fratricide, biofilm formation, virulence	[8,32]
*Vibrio harveyi*	HAI-1, CAI-1 AI-2	LuxLM/LuxN LuxP/LuxQ	Bioluminescence emission, symbiosis	[7–9,11,26]
